# Optimizing acquisition times for total-body positron emission tomography/computed tomography with half-dose ^18^F-fluorodeoxyglucose in oncology patients

**DOI:** 10.1186/s40658-022-00474-y

**Published:** 2022-07-08

**Authors:** Yibo He, Yushen Gu, Haojun Yu, Bing Wu, Siyang Wang, Hui Tan, Yanyan Cao, Shuguang Chen, Xiuli Sui, Yiqiu Zhang, Hongcheng Shi

**Affiliations:** 1 Shanghai Institute of Medical Imaging, Shanghai, 200032 China; 2 Department of Nuclear Medicine, Zhongshan Hospital, Fudan University, Shanghai, 200032 China; 3 Nuclear Medicine Institute of Fudan University, Shanghai, 200032 China

**Keywords:** Half-dose, Acquisition times, Total-body PET/CT, Image quality, Lesion detectability

## Abstract

**Background:**

The present study aimed to explore the boundary of acquisition time and propose an optimized acquisition time range for total-body positron emission tomography (PET)/computed tomography (CT) oncological imaging using half-dose (1.85 MBq/kg) ^18^F-fluorodeoxyglucose activity based on diagnostic needs.

**Methods:**

In this retrospective study based on a total-body PET system (uEXPLORER), an exploration cohort (October 2019–December 2019) of 46 oncology patients was first studied. The acquisition time for all patients was 15 min, and the acquired images were reconstructed and further split into 15-, 8-, 5-, 3-, 2-, and 1-min duration groups (abbreviated as G15, G8, G5, G3, G2, and G1). The image quality and lesion detectability of reconstructed PET images with different acquisition times were evaluated subjectively (5-point scale, lesion detection rate) and objectively (standardized uptake values, tumor-to-background ratio). In the same way, the initial optimized acquisition times were further validated in a cohort of 147 oncology patients (December 2019–June 2021) by using the Gs images (the images obtained using the 15- and 10-min acquisition times) as controls.

**Results:**

In the exploration cohort, the subjective scores for G1, G2, G3, G5, and G8 images were 2.0 ± 0.2, 2.9 ± 0.3, 3.0 ± 0.0, 3.9 ± 0.2, and 4.2 ± 0.4, respectively. Two cases in G1 were rated as 1 point. No significant difference in scores was observed between G5 and G8 (*p* > 0.99). In general, groups with a longer acquisition time showed lower background uptake and lesion conspicuity. Compared with G15, lesion detection rate significantly reduced to 85.3% in G1 (*p* < 0.05). In the validation cohort, the subjective score was 3.0 ± 0.2 for G2, 3.0 ± 0.1 for G3, 3.6 ± 0.5 for G5, 4.0 ± 0.3 for G8, and 4.4 ± 0.5 for Gs. Only the scores between G2 and G3 were not significantly different (*p* > 0.99). The detection rates (204 lesions) significantly reduced to 94.1–90.2% in G3 and G2 (all *p* < 0.05).

**Conclusion:**

A 2-min acquisition time provided acceptable performance in certain groups and specific medical situations. And protocols with acquisition times ≥ 5 min could provide comparable lesion detectability as regular protocols, showing better compatibility and feasibility with clinical practice.

**Supplementary Information:**

The online version contains supplementary material available at 10.1186/s40658-022-00474-y.

## Background

As a noninvasive medical imaging modality, positron emission tomography/computed tomography (PET/CT) plays a crucial role in cancer diagnosis, staging, restaging, and efficacy assessments. Currently, the most widely available PET/CT tracer is the ^18^F-labeled glucose analogue fluorodeoxyglucose (FDG) [1]. In routine clinical practice, only a small amount (≤ 1%) of the radiation emitted by the tracer injected into the patient’s body is detected during PET/CT due to the limited field of view (FOV) and the attenuation or scattering of a large portion of the photon pairs within the FOV [2]. Thus, high-quality PET images are generated either by increasing the injected dose of the tracer or by prolonging the acquisition time, both of which may result in health-and-safety consequences for patients.

Many researchers [3–5] have attempted to improve the sensitivity of PET. In 2019, a total-body PET/CT scanner (uEXPLORER, United Imaging Healthcare, China) with a 194-cm-long axial FOV was introduced to our center. It showed superior performance in clinical practice compared to other conventional PET/CT scanners in our center. This scanner was designed to have a superior sensitivity of 176 kcps/MBq and a high spatial resolution of 2.9 mm and can theoretically provide an approximately 40-fold sensitivity gain over other conventional PET scanners used for total-body imaging [6]. The improved sensitivity provides feasible opportunities for low-dose imaging or short-acquisition protocols.

Tan et al. demonstrated that total-body PET (uEXPLORER) with half-dose (1.85 MBq/kg) ^18^F-FDG activity and an acquisition time of 2 min could achieve a comparable image quality to conventional PET in lung cancer [7]. However, this result could not be directly extrapolated to other tumors, and a universally accepted optimal acquisition time does not yet exist. Referring to a previous study [8], we believe that a total acquisition time of 5 min could be used as the regular acquisition time for full-dose (3.7 MBq/kg) total-body PET imaging. Therefore, it is logical to presume that a 10-min acquisition time would be sufficient as the routine acquisition time for total-body PET imaging with half-dose FDG. In this study, we aimed to identify a suitable acquisition time range for half-dose total-body PET oncological imaging by analyzing several acquisition times and the quality of the resultant PET images.

## Materials and methods

### Patient selection

This retrospective study included two cohorts of patients: the initial exploration cohort and the clinical validation cohort. The initial exploration cohort consisted of 46 consecutive patients who underwent ^18^F-FDG total-body PET/CT with half-dose activity for oncological diagnosis and/or clinical staging in our department (from October to December 2019). This cohort was used to establish the preliminary optimization scheme of acquisition time. The second cohort included 147 patients (from December 2019 to June 2021) with malignancy or suspected malignancy who were selected retrospectively in an attempt to not only confirm the results of the first cohort but also to further identify the optimal range of acquisition times.

The inclusion and exclusion criteria were the same for both cohorts. The key inclusion criteria were patients with definite pathological diagnoses from a surgical specimen who were untreated before surgery. In addition, contrast-enhanced CT or contrast-enhanced magnetic resonance imaging was performed before surgery to rule out false-negative findings on PET images. The exclusion criteria were as follows: (1) relative preoperative treatments or no authentic pathological evidence, (2) diffuse lesions in the liver, (3) lesion biopsy with negative results, (4) the time delay between the injection and the image acquisition < 40 min or > 120 min, and (5) claustrophobia or other conditions causing poor cooperation. The study was approved by the institutional review board of Zhongshan Hospital, Fudan University, and informed consent was acquired from all enrolled patients.

### PET/CT examination

All patients fasted for at least 6 h before ^18^F-FDG administration (half dose: 1.85 MBq/kg), with a fluctuation of 20%. In the exploration cohort, raw images of all patients were obtained over a total acquisition time of 15 min on a 194-cm-long axial FOV total-body PET/CT scanner (uEXPLORER, United Imaging Healthcare, Shanghai, China) with the 3D list mode. The PET images of each patient were first reconstructed using the entire 15-min data and were further split into the 8-, 5-, 3-, 2-, and 1-min acquisition groups to mimic fast-acquisition scenarios. For brevity, the image series reconstructed with acquisition times of 15–1 min are abbreviated as G15, G8, G5, G3, G2, and G1. In the clinical validation cohort, data acquisition was performed for 15 or 10 min in the 3D list mode. The images obtained using the 15- and 10-min acquisition times are collectively referred to as Gs images. The reason for grouping these two types of images together is that in April 2020, the standard acquisition time in our department was set at 10 min for optimization purposes. To further explore the results of the exploration cohort, all PET images were reconstructed and further divided into Gs, G8, G5, G3, and G2 images.

All PET images were reconstructed using an ordered subset expectation maximization (OSEM) algorithm that incorporated high-resolution time-of-flight (TOF) and point-spread function (PSF) modeling, with a spectrum of parameters: 3 iterations; 20 subsets; matrix, 192 × 192; slice thickness, 1.443 mm; and FOV, 600 mm (voxel size, 3.125 × 3.125 × 1.443 mm^3^) with a Gaussian post-filter (3 mm). The acquisition parameters for diagnostic CT were as follows: tube current modulation; voltage, 120 kV; pitch, 0.9625; and reconstructed slice thickness, 1 mm with a slice increment of 1 mm. All image analysis was conducted using a commercial medical image processing workstation (uWS-MI, United Imaging Healthcare, Shanghai, China).

### Qualitative image analysis in both cohorts

**Fig. 1:**
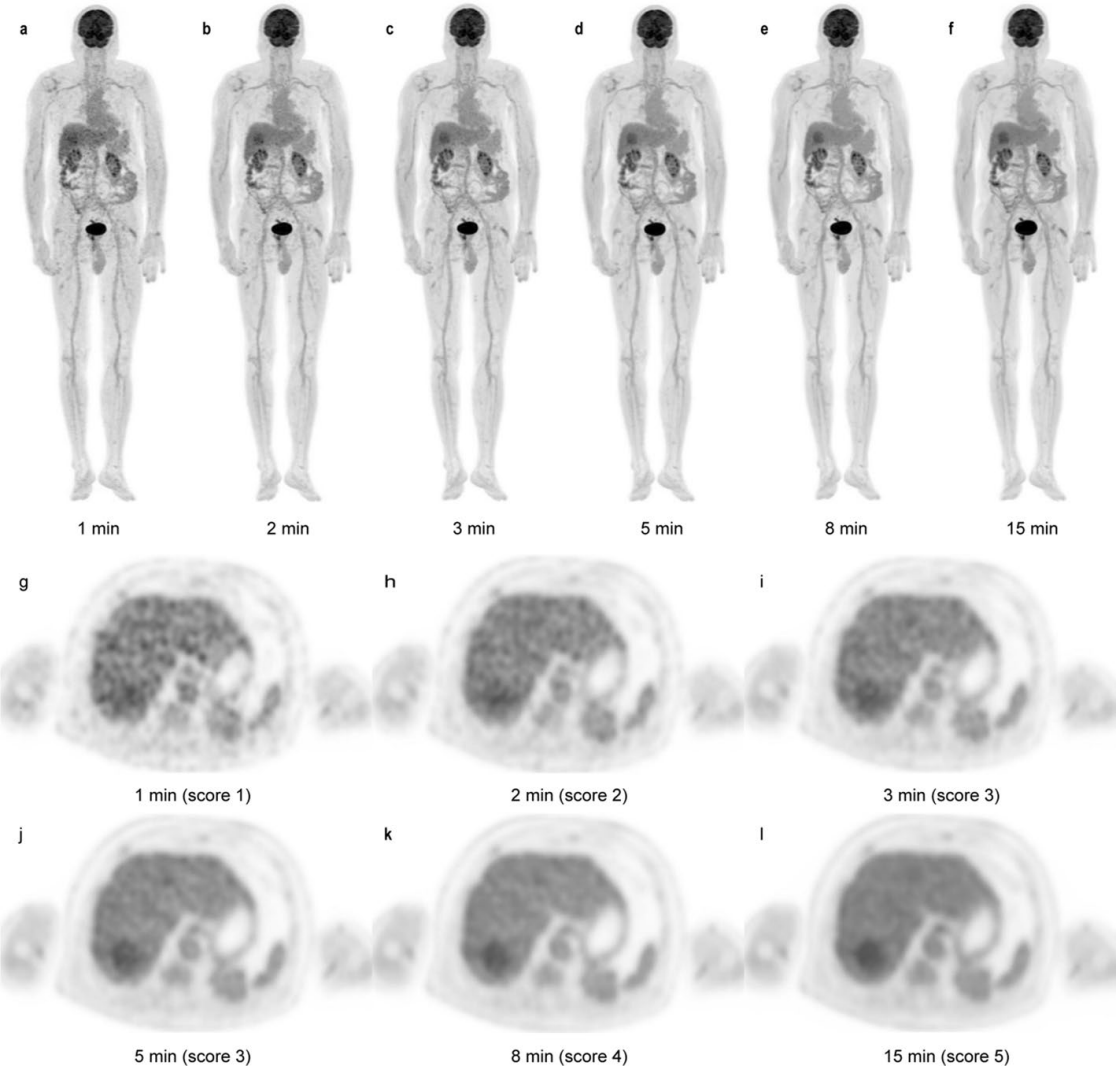
An example of qualitative analysis. An FDG-avid lesion with a diameter of 50.0 mm in the right posterior lobe of the liver is seen on G1 to G15 images with maximum intensity projection (MIP) (**a**–**f**) and the axial view (**g**–**l**). The axial view G1, G2, G3, G5, G8, and G15 images were given scores of 1, 2, 3, 3, 4, and 5, respectively

To identify an acceptable threshold of acquisition time, we performed a qualitative analysis of the reconstructed PET images from the exploration cohort. Before starting the assessments, two experienced nuclear radiologists were given the criteria for image grading (5-point Likert scale, Additional file 1: Table S1), as widely used in previous studies [9, 10]. After familiarizing themselves with the scoring method, the observers independently analyzed the image quality of the PET scans. Both readers were blinded to the medical history of each patient, injected dose, and acquisition time of the PET images. The order of image presentation was randomized to reduce the bias. The scoring of the images on the Likert scale was mainly based on the overall impression of the image quality, which was categorized as follows: 5 = excellent image quality, 4 = superior to the average image quality in routine practice in our department, 3 = equal to the quality used in clinical practice, 2 = acceptable image quality with no need to rescan, and 1 = non-diagnostic image requiring rescanning (Fig. 1). Subsequently, under the same dose regimen, the proposed acquisition durations were further validated in the clinical validation cohort. The subjective image quality in this cohort was rated independently using the same scoring criteria by the same qualified physicians. In cases of discrepancy, the PET images were discussed until a consensus was reached.

### Quantitative image analysis in the exploration cohort

The objective image quality measurements were conducted by an experienced technician under the supervision of a nuclear medicine physician. For measuring the background liver uptake, a two-dimensional circular ROI with a diameter of 20 mm was manually placed in the right lobe of the liver at the section of the portal vein bifurcation, avoiding all distinguishable lesions and intrahepatic large blood vessels. For measuring the background uptake of the mediastinal blood pool, the same-size ROI or an appropriately reduced ROI was plotted in the descending aorta at the level of the bronchial bifurcation, avoiding the vascular wall. Semiquantitative uptake parameters of the liver and mediastinal blood pool, the maximum standardized uptake value (SUV_max_), the mean SUV (SUV_mean_), and the standard deviation (SD) of the ROI were measured and documented. In addition, the liver signal-to-noise ratio (SNR) was calculated as the SUV_mean_ divided by its SD, and the liver coefficient of variation (COV) was obtained by dividing the SD by its SUV_mean_. All the same-size ROIs were simultaneously drawn on the same slice and site to minimize inter-site variation between all groups.

As representative indices of objective image quality as well as lesion conspicuity, the SUV_max_ and peak SUV (SUV_peak_) of the lesions and the tumor-to-background ratio (TBR) were included in the final analysis. Volumes of interest (VOIs) were manually placed on the lesions for SUV_max_ and SUV_peak_ measurements. To ensure that the entire lesion was covered, VOIs were placed at the largest diameter of the lesions on the transverse view. The TBR, which refers to lesion contrast, was calculated as the SUV_max_ of the lesion divided by the SUV_mean_ of the liver.

### Lesion detectability

The lesion detection rates for all groups of images were calculated to quantify the lesion detectability of the PET images from the two cohorts. The number of lesions identified in the G15 (exploration dataset) and Gs (validation dataset) images was selected as references. For each cohort, all PET images were read during a joint session by the same two nuclear medicine physicians, after an interval of 2 weeks since the qualitative analysis. The whole process of reading was in line with the principle of blinding and randomization to minimize any potential memory effect. Information of all ^18^F-FDG-avid lesions confidentially identified by the readers was documented and selected for the analysis. Images with poor quality or excessive background noise that made the lesion contrast unfavorable were marked. Discrepancies in lesion identification were resolved via mutual consultation.

### Statistical analysis

Statistical analysis and graph generation were conducted using SPSS 26.0 (IBM, SPSS) and Prism 8 (GraphPad Software Inc.), with *p* values < 0.05 being considered significant. Continuous variables were reported as mean ± SD. The Friedman’s test and Bonferroni-adjusted post hoc comparisons for multiple comparisons were applied to compare the results of subjective image scores and quantitative parameters among groups. Differences in lesion detection rate were evaluated using the Cochran’s Q test.

## Results

### Patient cohorts

The present study involved two patient cohorts: an initial exploration cohort for the analysis of different acquisition times and a clinical validation cohort for the validation of the initial results and the optimization of acquisition times. The results for these two cohorts are presented below.

### Initial exploration cohort

#### Patient characteristics

The demographic and clinical data of the patients are summarized in Table 1. We enrolled a total of 46 patients (34 men, 12 women) in the first cohort, with a mean age of 61.5 ± 8.8 years, a mean body mass index (BMI) of 24.1 ± 3.5 kg/m^2^, and a mean injected FDG dose of 1.88 ± 0.09 MBq/kg. The overall distribution of primary neoplasms in this cohort was as follows: lung neoplasms, 19 patients; colorectal cancer, 17 patients; liver cancer, 4 patients; biliary tract cancer, 2 patients; and stomach neoplasms, 4 patients. Lymph node metastases were suspected in 18 patients, while distant metastases were not present in any patient. The distribution of the primary tumors and lymph node lesions is shown in detail in Table 2.

**Table 1:** Characteristics of the patients in the exploration and validation cohorts

Patient characteristic	Initial exploration cohort (*n* = 46)	Clinical validation cohort (*n* = 147)
Gender (male/female)	34*/12*	79*/68*
Age (years)	61.5 ± 8.8 [43–81]	59.4 ± 12.1 [20–87]
Weight (kg)	67.0 ± 11.2 [46.3–90.7]	63.8 ± 11.7 [38.3–105.0]
Height (cm)	166.4 ± 7.4 [152.6–184.4]	164.3 ± 8.7 [139.0–185.2]
Blood glucose	5.7 ± 1.1 [4.1–9.1]	5.9 ± 1.4 [3.7–10.8]
BMI (kg/m^2^)	24.1 ± 3.5 [17.4–33.1]	23.6 ± 3.6 [15.5–36.0]
Injected dose (MBq)	125.9 ± 21.1 [85.1–174.4]	119.7 ± 22.3 [73.1–216.0]
Injected dose per unit weight (MBq/kg)	1.88 ± 0.09 [1.73–2.15]	1.88 ± 0.10 [1.67–2.18]
Uptake time (min)	64.7 ± 17.2 [40–106]	73.4 ± 17.5 [40–115]
Lesion counts (lesion/patient)	1.6 ± 0.8 [1–4]	1.6 ± 1.1 [1–8]
Lesion short diameter (mm)	15.8 ± 12.8 [4.9–85.5]	20.8 ± 18.6 [4.0–120.4]

Unless otherwise specified, data are presented as mean ± SD [range: min to max]

*Data are numbers of patients. BMI = body mass index

**Table 2:** Pathological distribution of primary tumors and suspected metastases in both cohorts

Pathological distribution	Primary tumor	Lymph node metastasis	Distant metastasis
*Exploration cohort*			
Lung (*n* = 19)	17/1/1 (22^†^)	2/0/0 (2^†^)	–
Biliary tract (*n* = 2)	2/0/0 (2^†^)	1/1/0 (3^†^)	–
Liver (*n* = 4)	3/1/0 (5^†^)	0/1/0 (2^†^)	–
Colorectum (*n* = 17)	17/0/0 (17^†^)	8/3/1 (17^†^)	–
Stomach (*n* = 4)	4/0/0 (4^†^)	1/0/0 (1^†^)	–
Total (*n* = 46)	50^†^	25^†^	0^†^
*Clinical validation cohort*			
Lung (*n* = 11)	11/0/0 (11^†^)	1/0/1 (4^†^)	–
Biliary tract (*n* = 30)	29/1/0 (31^†^)	11/4/0 (19^†^)	–
Liver (*n* = 19)	16/2/1 (23^†^)	2/1/0 (4^†^)	–
Colorectum (*n* = 10)	10/0/0 (10^†^)	3/3/2/1 (19^†^)	1/0/2 (7^†^)
Stomach (*n* = 4)	3/0/1 (6^†^)	2/0/0 (2^†^)	–
Pancreas (*n* = 26)	24/2/0 (28^†^)	5/0/0 (5^†^)	–
Gall bladder (*n* = 5)	5/0/0 (5^†^)	0/1/1 (5^†^)	–
Bladder (*n* = 7)	4/3/0 (10^†^)	–	–
Breast (*n* = 3)	3/0/0 (3^†^)	1/0/0 (1^†^)	1/0/0 (1^†^)
Ovary (*n* = 4)	3/1/0 (5^†^)	–	–
Small intestine (*n* = 9)	9/0/0 (9^†^)	2/0/0 (2^†^)	–
Pharynx (*n* = 3)	3/0/0 (3^†^)	0/0/0/0/0/0/1 (7^†^)	–
Skin (*n* = 1)	1/0/0 (1^†^)	1/0/0 (1^†^)	–
Others (*n* = 15)*	15/0/0 (15^†^)	–	–
Total (*n* = 147)	160^†^	69^†^	8^†^

Values separated by virgules (/) indicate the number of patients with 1/2/3/4/5/6/7 lesions

^†^Data are numbers of lesions

*Others (*n* = 15) include the following single primary tumors without metastasis: kidney (*n* = 4), esophagus (*n* = 2), mediastinum (*n* = 3), uterus (*n* = 1), bones (*n* = 1), carotid body (*n* = 1), muscle (*n* = 1), and retroperitoneum (*n* = 2)

#### Evaluation of image quality

As shown in Table 3, the average qualitative scores for images with acquisition times of 1, 2, 3, 5, and 8 min (henceforth referred to as G1, G2, G3, G5, and G8, respectively) were 2.0 ± 0.2, 2.9 ± 0.3, 3.0 ± 0.0, 3.9 ± 0.2, and 4.2 ± 0.4, respectively. Even for the G1 images, the average score indicated visually acceptable images with no need for rescanning. In the case of two patients, however, the G1 images were given a score of 1 point. The subjective scores of the G5 and G8 images were significantly higher than those of G1, G2, and G3 (all *p* < 0.05). No significant difference in subjective scores was observed between the G5 and G8 images (*p* > 0.99). In addition, no distinct difference was identified between the G2 and G3 images (*p* > 0.99).

**Table 3:** Qualitative image analysis in the exploration and validation cohorts

Group	Excellent (score 5)	Good (score 4)	Average (score 3)	Acceptable (score 2)	Non-diagnostic (score 1)	Average score
*Exploration cohort*						
G1 (*n* = 46)	0	0	0	44 (95.7%)	2 (4.3%)	2.0 ± 0.2*
G2 (*n* = 46)	0	0	40 (87.0%)	6 (13.0%)	0	2.9 ± 0.3*
G3 (*n* = 46)	0	0	46 (100%)	0	0	3.0 ± 0.0*
G5 (*n* = 46)	0	43 (93.5%)	3 (6.5%)	0	0	3.9 ± 0.2*
G8 (*n* = 46)	10 (11.7%)	36 (78.3%)	0	0	0	4.2 ± 0.4*
*Clinical validation cohort*						
G2^†^ (*n* = 147)	0	0	143 (97.3%)	4 (2.7%)	0	3.0 ± 0.2*
G3^†^ (*n* = 147)	0	1 (0.7%)	146 (99.3%)	0	0	3.0 ± 0.1*
G5^†^ (*n* = 147)	0	85 (57.8%)	62 (42.2%)	0	0	3.6 ± 0.5*
G8^†^ (*n* = 147)	8 (5.4%)	134 (91.2%)	5 (3.4%)	0	0	4.0 ± 0.3*
Gs^†^ (*n* = 147)	63 (42.9%)	84 (57.1%)	0	0	0	4.4 ± 0.5*

Unless otherwise indicated, data are numbers of patients, and data in parentheses are percentages

*Data are mean ± standard deviation (mean ± SD)

^†^Groups from the clinical validation cohort

**Fig. 2:**
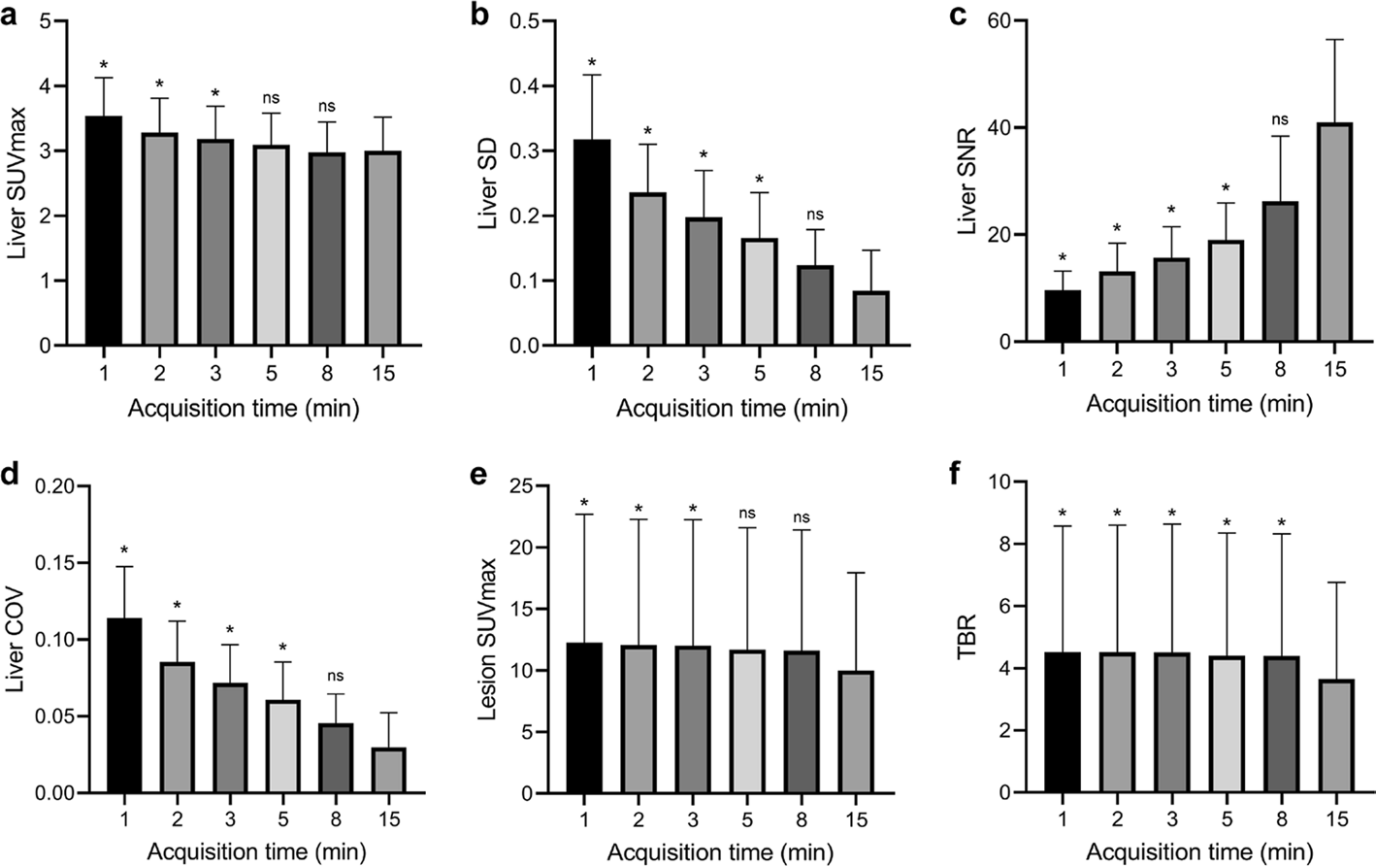
Box plots for comparison of quantitative parameters in liver (**a**–**d**) and lesion (**e**–**f**) among groups. In general, the liver uptake and image noise decreased as the acquisition time increased, while the SNR of liver gradually increased. G15 served as control; there were no significant differences between G8 and G15 for any of these parameters (**a**–**d**). For lesions, SUV_max_ (**e**) was significantly lower on G15 images than on G3, G2, and G1 images, but did not differ significantly between G15 and G8 (*p* > 0.99) or between G15 and G5 (*p* = 0.35). The TBR (**f**) significantly differed between short-duration images and G15 images. SUV = standardized uptake value, SD = standard deviation, SNR = signal-to-noise ratio, COV = coefficient of variation, TBR = tumor-to-background ratio (^*^indicate *p* < 0.05; ns, not significant)

The results of the objective image quality assessments are presented in Table 4 and Fig. 2a–d. In general, the background uptake and image noise decreased as the acquisition time increased, while the liver SNR gradually increased. For the liver uptake, there were no significant differences in SUV_max_ between G5, G8, and G15 (all *p* ≥ 0.16, G15 served as control). The liver SD, SNR, and COV in G15 images were not significantly different from those of G8 images (all *p* ≥ 0.10), while they differ significantly from those of the other short-duration images (all *p* < 0.05). For the blood pool uptake, the SUV_max_ was significantly lower in the G15 images than in the G3, G2, and G1 images (all *p* < 0.05), but did not differ between the G15 and G8 images (*p* > 0.99) or between the G15 and G5 images (*p* = 0.55). The average SD of the regions of interest (ROIs) in the blood pool significantly differed between the G15 images and the other images (all *p* < 0.05).

**Table 4:** Quantitative parameters of the background and lesions in the exploration cohort

Measurement	G15	G8	G5	G3	G2	G1
Liver SUVmax^†^	3.00 ± 0.52	2.98 ± 0.47	3.09 ± 0.49	3.19 ± 0.50*	3.28 ± 0.53*	3.53 ± 0.60*
Liver SD^†^	0.084 ± 0.063	0.124 ± 0.055	0.166 ± 0.070*	0.198 ± 0.072*	0.236 ± 0.074*	0.317 ± 0.100*
Liver SNR^†^	40.98 ± 15.48	26.22 ± 12.19	19.02 ± 6.89*	15.70 ± 5.79*	13.16 ± 5.25*	9.66 ± 3.56*
Liver COV^†^	0.030 ± 0.023	0.046 ± 0.019	0.061 ± 0.025*	0.072 ± 0.025*	0.086 ± 0.027*	0.114 ± 0.033*
Mediastinal SUVmax^†^	2.27 ± 0.37	2.30 ± 0.37	2.33 ± 0.40	2.41 ± 0.43*	2.49 ± 0.45*	2.61 ± 0.50*
Mediastinal SD^†^	0.118 ± 0.049	0.144 ± 0.047*	0.154 ± 0.039*	0.171 ± 0.045*	0.196 ± 0.054*	0.249 ± 0.083*
Lesion SUVmax^†^	9.99 ± 7.94	11.60 ± 9.82	11.68 ± 9.92	12.01 ± 10.25*	12.06 ± 10.23*	12.27 ± 10.42*
Lesion SUVpeak^†^	8.27 ± 6.46	9.01 ± 7.40	8.99 ± 7.36	9.01 ± 7.41	9.07 ± 7.42	8.98 ± 7.51
TBR^†^	3.65 ± 3.11	4.39 ± 3.93*	4.39 ± 3.96*	4.51 ± 4.13*	4.51 ± 4.09*	4.51 ± 4.06*

The measured sample sizes for the background and lesion were 46 and 56, respectively

*SUVmax* maximum standardized uptake value; *SD* standard deviation; *SNR* signal-to-noise ratio; *COV* coefficient of variation; *SUVpeak* peak standardized uptake value; *TBR* tumor-to-background ratio.

^†^Data are presented as mean ± standard deviation (mean ± SD)

*Significant difference in comparison with the control group (G15), *p* < 0.05

#### Lesion detectability

Pathological examination confirmed a total of 75 lesions in the 46 patients. Of these, 7 lesions in 6 patients (2 liver lesions, 1 lung lesion, and 4 lymph node lesions) were not recognizable on G15 images. In all, 47 primary lesions and 21 suspicious lymph node metastases were detected on G15 images. G15 served as control; the lesion detection rates were 85.3% (58/68) and 97.1% (66/68) for the G1 and G2 images, respectively, and 100% (68/68) for the remaining images. On the G1 images, 10 lesions from 6 patients were not identifiable, including 1 lesion in the liver and 9 lesions in the lymph nodes. The lesion detection rate significantly differed between the G15 and G1 images (*p* < 0.05).

For the assessment of lesion conspicuity, a total of 56 out of 75 lesions were pathologically malignant and included for analysis (Table 4 and Fig. 2e, f). The lesion SUV_max_ was significantly lower on G15 images than on G3, G2, and G1 images, but did not differ significantly between G15 and G8 (9.99 ± 7.94 vs*.* 11.60 ± 9.82; *p* > 0.99) or between G15 and G5 (9.99 ± 7.94 vs*.* 11.68 ± 9.92; *p* = 0.35). In addition, the corresponding TBRs of the lesions on G15 images were lower than all the short-duration images (all *p* < 0.05). No significant difference in lesion SUV_peak_ was found between G15 and the short-duration images. Detailed SUVs and TBRs of different primary tumors and metastatic lymph nodes are summarized in Additional file 2: Table S2.

### Clinical validation cohort

#### Patient characteristics

A total of 147 eligible patients (79 men, 68 women) with a mean age of 59.4 ± 12.1 years, mean body weight of 63.8 ± 11.7 kg, mean BMI of 23.6 ± 3.6 kg/m^2^, and mean injected FDG dose of 1.88 ± 0.10 MBq/kg were included in this cohort (Table 1). After integrating the pathological data, we included 240 lesions in the final analysis: 163 primary tumors, 69 suspicious lymph node metastases, and 8 distant metastases. Three patients had multiple primary tumors: one patient had small intestine cancer with schwannomas; one had bladder cancer with ureteral cancer; and another patient had liver cancer with gastrointestinal stromal tumor. Distant metastases were present in 4 patients, including 3 patients with colorectal cancer and liver metastasis and 1 patient with breast cancer and bone metastasis. The distribution of all pathologically confirmed lesions in the validation cohort is shown in detail in Table 2.

#### Subjective image quality

As presented in Table 3, the subjective scores for the G2, G3, G5, G8, and Gs (acquisition time, 10 or 15 min) images were 3.0 ± 0.2, 3.0 ± 0.1, 3.6 ± 0.5, 4.0 ± 0.3, and 4.4 ± 0.5, respectively. The scores for the G2 and G3 images were approximately 3 points or slightly lower than 3 points. Significant differences in these scores were observed between any two groups (all *p* < 0.05), except for G2 and G3 (*p* > 0.99).

#### Lesion detectability

Of the 240 lesions, 36 lesions were not clearly identified on Gs images: 11 liver lesions, 5 biliary tract lesions, 2 pancreatic lesions, 2 bladder lesions, 1 gallbladder lesion, and 15 lymph node lesions. Compared to the Gs images, the G2, G3, G5, and G8 images had lesion detection rates of 90.2% (184/204), 94.1% (192/204), 99.0% (202/204), and 100% (204/204), respectively. The distribution of the 20 lesions that went undetected on G2 images was as follows: biliary tract (*n* = 3), liver (*n* = 2), pancreas (*n* = 1), stomach (*n* = 1), bladder (*n* = 1), small intestine (*n* = 1), lymph nodes (*n* = 10), and liver metastasis (*n* = 1). The distribution of lesions that were unidentifiable on G3 images was as follows: biliary tract (*n* = 2), bladder (*n* = 1), liver (*n* = 1), and lymph nodes (*n* = 8). The lesion detection rates for the G5 and G8 images were not significantly lower than the rates for the Gs images (all *p* > 0.99).

## Discussion

The predicted 40-fold gain in sensitivity of the total-body PET compared to conventional PET scans would offer a wide range of combinations of injection doses and scan times [11, 12], providing the foundations for the creation of scans with credible image quality based on clinical needs and the need for different acquisition speeds, which was the theoretical basis of this study.

**Fig. 3:**
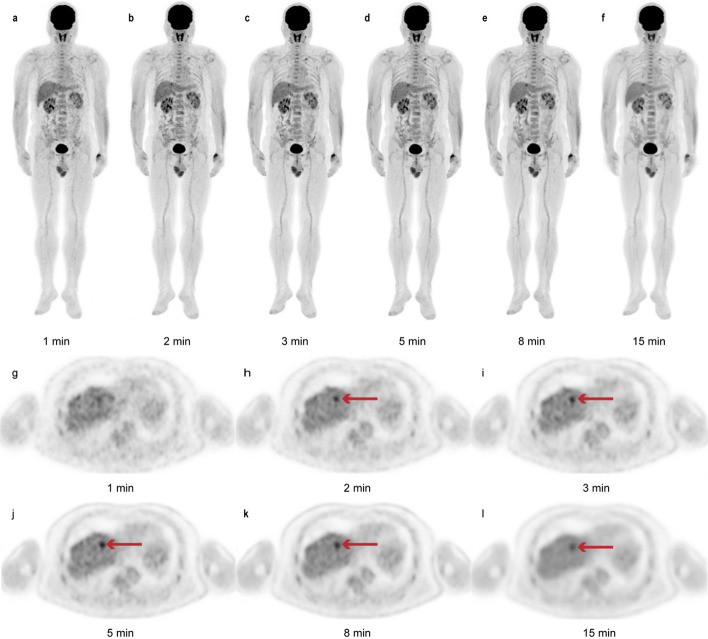
A 56-year-old man with intrahepatic cholangiocarcinoma. The images show a lesion on G1 to G15 images with maximum intensity projection (MIP) (**a**–**f**) and the axial view (**g**–**l**). As indicated by the red arrowheads, the lesion is clearly identified on the G2, G3, G5, G8, and G15 images, but cannot be distinguished from background noise when the acquisition time is reduced to 1 min (G1 images)

At a constant injected dose, a higher image quality always requires a longer acquisition time [13]. To determine the acceptable threshold of acquisition time at half-dose FDG, we conducted a preliminary exploration study. The results indicated that the subjective quality scores of the G1, G2, and G3 images were significantly inferior to those of the G5 and G8 images (all *p* < 0.05). Nevertheless, the subjective quality scores of G1 (2.0 ± 0.2) and G2 (2.9 ± 0.3) images were acceptable, though these images provided limited clinical information. Only reconstructed PET images with acquisition times of 3 min or longer received subjective scores that are defined as appropriate for routine clinical application in our department (mean scores for G3, G5, and G8 images: 3.0 ± 0.0, 3.9 ± 0.2, and 4.2 ± 0.4, respectively). Notably, for 2 patients, the G1 images received a score of only 1 point, which indicated that the images were non-diagnostic, and the patients had to be rescanned. The objective quality analysis showed that the SUVs of the liver and mediastinal blood pool gradually decreased with an increase in the acquisition time, in the order of G1, G2, G3, G5, G8, and G15; this is consistent with the results reported by Zhang et al. [9]. This finding suggested that the extension of acquisition time could reduce image noise and permit further reduction in the SUVs of the liver and mediastinal blood pool, thereby enhancing the image contrast [14]. Furthermore, our results showed no significant differences in SUV_max_ between G5, G8 and G15, whether in the liver, the blood pool, or the lesion (all *p* ≥ 0.16). Considering that SUV_max_ is widely used in clinical PET imaging, we speculated that protocols with acquisition times ≥ 5 min may provide sufficient parameters for clinical applications. Meanwhile, as shown in Fig. 2e, f, we noted that both lesion SUV_max_ and TBR followed a similar pattern of variation, and that the differences in the average values of SUV_max_ and TBR for G15 and each short-acquisition groups were greater than the differences between the short-acquisition groups. We believed that there were two main factors explaining this phenomenon. Firstly, from a clinical point of view, the results of this dataset were vulnerable to the tumors that made up the majority due to the context of multimorbidity and limited sample size of the patients in this cohort. Based on statistics classified according to primary tumor site, we could find that this phenomenon was pronounced in gastrointestinal tumors. As patients with gastrointestinal malignancies are often accompanied by inflammatory changes such as gastrointestinal ulcers, this makes it impossible to accurately distinguish between tumor parenchyma and inflammatory lesions when outlining the tumor. Therefore, we speculated that the possible high SUV_max_ and TBR of inflammatory lesions decreased with time, with changes becoming more pronounced the longer the acquisition time. And, the SUV_max_ and TBR of G15 may indicate estimates closer to the true metabolism of tumor parenchyma. Secondly, for lesions of different sizes, not only will the SUV_max_ be in a different range, but the variation pattern of the quantitative parameters over the acquisition time will also be different [15, 16]. In general, the smaller the lesion, the more its SUV_max_ is affected by the acquisition time; however, the absolute value of the SUV_max_ of a small lesion is usually smaller than that of a large lesion at the same contrast [16]. This study did not disaggregate the statistics by lesion size, but rather integrated the quantitative parameters for all lesions of all sizes, which could have affected the statistical results. Additionally, with the G15 images as the reference, the lesion detection rate was 100% for all images, except for the G1 (85.3%, *p* < 0.05) and G2 (97.1%, *p* > 0.99) images. A drawback of the short acquisition time is an increase in noise caused by low photon counts, which ultimately results in false-positive lesions on visual PET image assessment. Thus, some of the positive lesions identified using short acquisition times possibly represent false-positive lesions, as was typical for the G1 images (Fig. 3). However, we did not observe any differences in the lesion detection rates of the G3, G5, and G8 images, possibly due to the small sample size and selection bias. Although the shortened acquisition time exhibited higher SUVs and TBRs of the lesions in the exploration cohort, its tendency to result in impaired image quality and indistinguishable false-positive lesions indicate the need for a further optimized acquisition time.

**Fig. 4:**
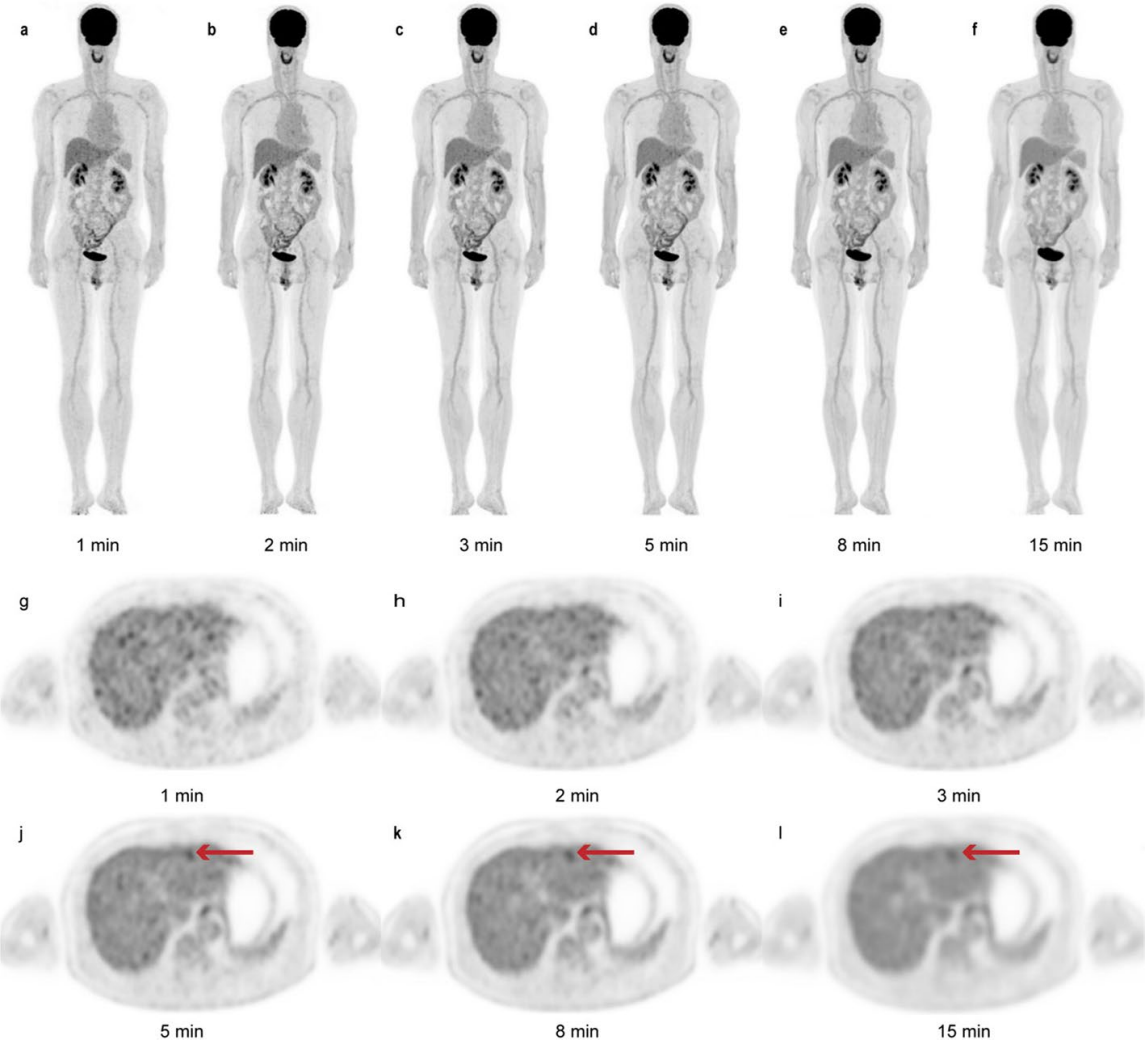
A 63-year-old man with hepatocellular carcinoma. The images show an FDG uptake lesion in the left external lobe of the liver on G1 to G15 images with maximum intensity projection (MIP) (**a**–**f**) and the axial view (**g**–**l**). The background noise is notably higher on the G1, G2, and G3 images, which has resulted in impaired image quality and low confidence of lesion detectability. Thus, the lesion in the liver (arrows in **j**–**l**) was masked by the background noise when the acquisition time was 3 min or lower

Our preliminary results were further validated in a dataset with a larger sample size. The subjective image quality analysis in the validation dataset indicated that the scores for the G2 and G3 images (3.0 ± 0.2 vs*.* 3.0 ± 0.1; *p* > 0.99) were significantly lower than those for the other images (all *p* < 0.05). The subjective G2 and G3 scores were only 3 points or slightly lower than 3 points, which is the minimum score required for routine clinical practice in our department. We speculated that one reason for the impaired image quality in some patients was the BMI (up to 36.0 kg/m^2^). PET image quality is influenced by several factors, including BMI [17–20], which were not strictly controlled for in the patients enrolled in our study. Thus, this patient group was, to some extent, a reflection of the real clinical situation. Compared to the Gs images, the G8 and G5 images showed lesion detection rates of 100% and 99.0%, respectively, while the G3 and G2 images showed significantly lower lesion detection rates (94.1% and 90.2%, respectively; all *p* < 0.05). Consistent with the trends we found, a previous study performed on an anthropomorphic thoracic phantom by Matheoud et al. [21] indicated that the detection performances decreased as the acquisition time decreased. In addition, a full-dose (4.4 MBq/kg) clinical study based on the total-body PET scanner (uEXPLORER) by Zhang et al. [9] showed that the detection rate was 100% in the 180-, 120-, and 60-s acquisition group and reduced to 91.7–78% in the group 30 and 18 s compared to the standard group (900 s). Thus, we concluded that a protocol with an acquisition time of 5 min or longer could provide comparable lesion detectability as the regular protocol. This result also reflected the differences between G2 or G3 and G5 or G8, providing a new perspective for the rational assessment of the SUVs and TBRs of the lesions in the exploration cohort. Unlike the G2 and G3 scans, the G5 and G8 images exhibited a high degree of lesion conspicuity without a significant compromise in image quality (Fig. 4). Nevertheless, it should be noted that some lesions were still missed at an acquisition time of 5 min, such as hilar cholangiocarcinoma (HC). Although HC is a highly malignant tumor where FDG theoretically tends to accumulate, liver uptake is often not significantly lower than the tumor uptake in most clinical cases. Lee et al. have reported a low cutoff SUV_max_ of 3.65 for differentiating HC from benign tumors [22]. This result might give some hints about individualizing the acquisition time appropriately for certain hypometabolic tumors in clinical practice.

On the basis of the results from the two cohorts, we propose a 5–8-min acquisition time range as an optimized universal scheme for the clinical application of total-body PET/CT at half-dose FDG, which is within the recommended range (3–10 min) proposed by Sui et al. [23]. Indeed, our study showed a gradient of acquisition times for different clinical needs. An acquisition time of 2 min was sufficient to obtain clinically acceptable image quality, while acquisition times of 3–5 min were adequate for most clinical applications (e.g., gastrointestinal neoplasms). In some cases, particularly for the liver, an acquisition time of 5–8 min was preferable to obtain more reliable information for disease assessment. It is worth noting that an acquisition time of 8 min not only resulted in high image quality but also served as an alternative to the 10-min protocol in terms of lesion detectability. The 2-min acquisition protocol we identified and validated was required for some specific occasions for two reasons. The first reason is that this protocol has a reasonably low radiation dose, which helps to decrease radiation exposure from medical procedures for medical staff. The European Association of Nuclear Medicine (EANM) guidelines recommend a minimum acceptable administered dose of 7 MBq·min·bed^−1^ kg^−1^ (with a PET bed overlap of > 30%) for ^18^F-FDG PET/CT oncological examination in adults [1], which means that it usually depends on the injected dose per unit weight, acquisition time, and the sensitivity profile of the PET bed overlap. Zhang et al. [9] reported a dose of 4.67 MBq min kg^−1^ for a 60-s acquisition time at full dose, which was already lower than the recommended minimum acceptable dose. In this study, the average injected dose was approximately 1.88 MBq/kg. After multiplied by 2-min scan duration, it becomes 3.76 MBq min kg^−1^ which is significantly lower than the recommendation dose (7 MBq min kg^−1^) and the value (4.67 MBq min kg^−1^) proposed by Zhang et al. [9]. This reduction in the injected dose would enable the performance of half-dose PET imaging for numerous applications in vulnerable groups (e.g., pregnant women) and patients requiring repeat PET examinations [24]. The second reason for the 2-min acquisition protocol was that the total scan time was significantly reduced. For a standard “skull to mid-thighs” whole-body tumor-imaging protocol at full-dose FDG, the PET acquisition takes about 10–20 min for the body part and approximately 3 min for the head [25]. The acquisition time typically varies according to the centers preference for lower dose or higher throughput scans, patient BMI, etc. [25]. In contrast, the present study showed that half-dose total-body PET/CT can be performed in just one bed position or, if necessary, in multiple bed positions using an acquisition time of only 2 min per bed position, which is only valid for the uEXPLORER scanner; this protocol is recommended for patients with unstable physical conditions, as it would greatly improve patient comfort.

Our study had several limitations. First, a selection bias might exist because only patients with diagnoses confirmed by postoperative pathology were included in our study. Second, as this was a single-center retrospective study, the results should be externally validated in a larger multicenter trial to further reduce selection bias. Finally, based on our experience, the lesion detection rate is affected by the size, shape, volume, and surroundings of the lesion as well as the readers’ experience. These intriguing factors warrant further investigation.

## Conclusion

Total-body PET/CT with half-dose (1.85 MBq/kg) FDG in oncology patients allows for a significant reduction in acquisition time with comparable image quality and lesion detectability to the regular acquisition protocol. A 2-min acquisition, though somewhat lowering quality, provided acceptable performance and warrants consideration in certain groups and specific medical situations. However, a 5–8-min acquisition time range demonstrates better compatibility and feasibility with clinical practice, providing sufficient information to meet the needs of clinical diagnosis.

## Supplementary Information

**Additional file 1:** **Table S1**. Scoring for subjective PET image quality by the Likert scale

**Additional file 2**: **Table S2**. Detailed SUVs and TBRs of different primary tumors and metastatic lymph nodes in the exploration cohort (*n* = 56)

## Data Availability

The data that support the findings of this study are available from the corresponding author upon reasonable request.

## Abbreviations

FDG: Fluorodeoxyglucose
PET/CT: Positron emission tomography/computed tomography
FOV: Field of view
OSEM: Ordered subset expectation maximization
TOF: Time of flight
PSF: Point spread function
Gs: Standard group
ROI: Region of interest
VOI: Volume of interest
SUV_max_: Maximum standardized uptake value
SUV_mean_: Mean standardized uptake value
SD: Standard deviation
SUV_peak_: Peak standardized uptake value
SNR: Signal-to-noise ratio
COV: Coefficient of variation
TBR: Tumor-to-background ratio
BMI: Body mass index
EANM: European Association of Nuclear Medicine
