# Supplementary material for: Optimizing acquisition times for total-body positron emission tomography/computed tomography with half-dose 18F-fluorodeoxyglucose in oncology patients
Source: EJNMMI Phys. 2022 Jul 8;9:45. doi: 10.1186/s40658-022-00474-y (PMC9270529; doi:10.1186/s40658-022-00474-y)
Supplement: Supplementary file 1 — Additional file 1: Table S1. Scoring for subjective PET image quality by the Likert scale [file 40658_2022_474_MOESM1_ESM.docx]

| **Supplementary Table 1** Scoring for subjective PET image quality by the Likert scale | | | |
| --- | --- | --- | --- |
| Grade | Overall image quality | Image noise | Lesion conspicuity |
| 1 | non-diagnostic | excessive noise | faliure to recognize |
| 2^*^ | acceptable | higher than the regular uM780 images | limited lesion delineation, impaired diagnostic confidence |
| 3 | equivalent to the regular uM780 images | similar to the regular uM780 images | recognizable with adequate information for diagnosis |
| 4 | superior to the average | lower than the regular uM780 images | fairly-displayed |
| 5 | excellent | near-unnoticeable noise | Well-defined, sharp lesion demarcation |

^*^ Subjective image score ≥ 2 points indicated a visually and diagnostically acceptable image with no need for rescanning.
