# Supplementary material for: Optimizing acquisition times for total-body positron emission tomography/computed tomography with half-dose 18F-fluorodeoxyglucose in oncology patients
Source: EJNMMI Phys. 2022 Jul 8;9:45. doi: 10.1186/s40658-022-00474-y (PMC9270529; doi:10.1186/s40658-022-00474-y)
Supplement: Supplementary file 2 — Additional file 2: Table S2. Detailed SUVs and TBRs of different primary tumors and metastatic lymph nodes in the exploration cohort (n = 56) [file 40658_2022_474_MOESM2_ESM.docx]

| **Supplementary Table 2** Detailed SUVs and TBRs of different primary tumors and metastatic lymph nodes in the exploration cohort (n = 56) | | | | | | |
| --- | --- | --- | --- | --- | --- | --- |
| Measurement | G15 | G8 | G5 | G3 | G2 | G1 |
| Lesion SUV_max_ (n=56) | 9.99 [1.03; 39.10] | 11.60 [1.10; 46.44] | 11.68 [1.18; 45.68] | 12.01 [1.18; 47.40] | 12.06 [1.13; 49.54] | 12.27 [1.18; 50.31] |
| Lung (n = 19) | 8.65 [1.03; 25.80] | 8.60 [1.16; 24.89] | 8.57 [1.18; 24.14] | 8.57 [1.18; 24.49] | 8.58 [1.13; 23.41] | 8.58 [1.18; 23.52] |
| Liver (n = 5) | 5.52 [3.17; 9.85] | 5.69 [3.14; 11.20] | 5.79 [3.41; 10.79] | 6.06 [3.54; 12.01] | 6.53 [3.60; 12.33] | 6.93 [3.87; 13.24] |
| Colorectum (n = 17) | 16.04 [5.57; 39.10] | 20.80 [7.91; 46.44] | 20.95 [7.48; 45.68] | 21.84 [7.42; 47.40] | 21.73 [7.78; 49.54] | 22.16 [6.66; 50.31] |
| Stomach (n = 4) | 10.30 [2.19; 26.65] | 12.54 [2.77; 33.34] | 13.16 [3.15; 34.52] | 13.42 [2.61; 34.23] | 13.59 [2.69; 34.62] | 13.75 [2.30; 34.89] |
| Biliary tract (n = 2) | 10.33 [9.13; 11.53] | 10.08 [8.54; 11.61] | 10.20 [8.74; 11.66] | 10.62 [9.05; 12.18] | 11.22 [9.31; 13.13] | 12.00 [10.10; 13.89] |
| Lymph nodes (n = 9) | 3.68 [1.10; 7.25] | 3.79 [1.10; 8.38] | 3.68 [1.24; 8.49] | 3.70 [1.33; 8.53] | 3.73 [1.15; 8.58] | 3.74 [1.24; 8.46] |
| Lesion SUV_peak_ (n=56) | 8.27 [0.90; 29.96] | 9.01 [0.52; 34.70] | 8.99 [0.52; 33.93] | 9.01 [0.55; 34.85] | 9.07 [0.54; 33.97] | 8.98 [0.68; 34.57] |
| Lung (n = 19) | 7.07 [0.97; 19.60] | 7.02 [1.04; 18.98] | 6.97 [1.04; 18.55] | 6.96 [1.02; 18.71] | 6.97 [0.94; 18.45] | 6.89 [0.93; 18.41] |
| Liver (n = 5) | 5.25 [3.02; 9.24] | 5.22 [2.93; 9.71] | 5.26 [3.12; 9.53] | 5.37 [3.17; 10.07] | 5.56 [3.16; 10.18] | 5.70 [3.33; 10.33] |
| Colorectum (n = 17) | 13.13 [4.59; 29.96] | 15.49 [5.40; 34.70] | 15.45 [5.10; 33.93] | 15.59 [5.00; 34.85] | 15.58 [5.26; 33.97] | 15.42 [4.57; 34.57] |
| Stomach (n = 4) | 8.62 [1.93; 21.45] | 9.55 [2.06; 24.32] | 9.79 [2.41; 24.41] | 9.56 [1.88; 23.09] | 9.91 [2.19; 24.49] | 9.87 [1.88; 24.56] |
| Biliary tract (n = 2) | 9.55 [8.47; 10.62] | 9.29 [7.85; 10.72] | 9.40 [7.98; 10.81] | 9.48 [8.12; 10.84] | 9.83 [8.37; 11.29] | 10.14 [8.63; 11.64] |
| Lymph nodes (n = 9) | 2.86 [0.90; 5.99] | 2.76 [0.52; 5.78] | 2.69 [0.52; 5.74] | 2.59 [0.55; 5.89] | 2.60 [0.54; 5.83] | 2.42 [0.68; 5.52] |
| TBR (n=56) | 3.65 [0.39; 15.16] | 4.39 [0.45; 17.59] | 4.39 [0.45; 17.50] | 4.51 [0.44; 18.30] | 4.51 [0.42; 18.28] | 4.51 [0.47; 17.65] |
| Lung (n=19) | 3.13 [0.39; 8.64] | 3.18 [0.45; 8.44] | 3.15 [0.45; 8.41] | 3.09 [0.44; 8.17] | 3.07 [0.42; 8.18] | 3.07 [0.47; 7.96] |
| Liver (n=5) | 1.65 [1.13; 2.90] | 1.85 [1.27; 3.58] | 1.85 [1.39; 3.28] | 1.95 [1.47; 3.61] | 2.09 [1.46; 3.88] | 2.18 [1.55; 3.98] |
| Colorectum (n=17) | 6.14 [2.59; 15.16] | 8.17 [3.57; 17.59] | 8.21 [3.32; 17.50] | 8.58 [3.11; 18.30] | 8.51 [3.04; 18.28] | 8.51 [2.77; 17.65] |
| Stomach (n=4) | 3.59 [0.87; 8.94] | 4.58 [1.10; 11.91] | 4.72 [1.24; 12.03] | 4.83 [1.04; 11.97] | 4.94 [1.07; 12.54] | 4.72 [0.92; 11.55] |
| Biliary tract (n=2) | 3.10 [2.95; 3.24] | 3.35 [3.12; 3.58] | 3.30 [3.13; 3.47] | 3.44 [3.15; 3.72] | 3.71 [3.26; 4.16] | 4.03 [3.27; 4.79] |
| Lymph nodes (n=9) | 1.28 [0.47; 2.48] | 1.36 [0.49; 3.12] | 1.32 [0.53; 3.14] | 1.32 [0.60; 3.28] | 1.35 [0.53; 3.38] | 1.32 [0.53; 3.09] |

SUVmax = maximum standardized uptake value, SUVpeak = peak standardized uptake value, TBR = tumor-to-background ratio (calculated as the lesion SUVmax divided by the liver SUVmean). Data are represented as mean [range: min to max].
